# Distance-based training in two community health centers to address tobacco smoke exposure of children

**DOI:** 10.1186/1471-2431-13-56

**Published:** 2013-04-17

**Authors:** Bethany Hipple, Emara Nabi-Burza, Nicole Hall, Susan Regan, Jonathan P Winickoff

**Affiliations:** 1 Center for Child and Adolescent Health Research and Policy, Massachusetts General Hospital, 50 Staniford Street Suite 901, Boston, MA 02144, USA; 2 AAP Richmond Center of Excellence, American Academy of Pediatrics, Elk Grove Village, IL, USA; 3 Tobacco Research and Treatment Center, Massachusetts General Hospital, Boston, MA, USA

**Keywords:** Tobacco smoke exposure, Secondhand smoke, Tobacco control, Online education, Community health centers, Parental smoking, Tobacco cessation

## Abstract

**Background:**

The CEASE (Clinical Effort Against Secondhand Smoke Exposure) intervention was developed to help pediatricians routinely and effectively address the harms of family smoking behaviors. Based on paper versions of CEASE, we partnered with the American Academy of Pediatrics’ online education department and developed a completely distance-based training, including an online CME training, handouts and education materials for families, and phone and email support.

**Methods:**

The pediatric offices of two low income health clinics with primarily Medicaid populations were selected for the study. Pre and post intervention data by survey of the parents was collected in both practices (Practice 1 n = 470; Practice 2 n = 177). The primary outcome for this study was a comparison of rates of clinician’s asking and advising parents about smoking and smoke-free home and cars.

**Results:**

Exit surveys of parents revealed statistically significant increases in rates of clinicians asking about parental smoking (22% vs. 41%), smoke-free rules (25% vs. 44%), and asking about other smoking household members (26% vs. 48%).

**Conclusions:**

Through a completely distance based intervention, we were able to train pediatricians who see low income children to ask parents about smoking, smoke-free home and car rules, and whether other household members smoke. Implementing a system to routinely ask about family tobacco use and smoke-free home and car rules is a first step to effectively addressing tobacco in a pediatric office setting. By knowing which family members use tobacco, pediatricians can take the next steps to help families become completely tobacco-free.

**Trial registration:**

Clinical trials number: NCT01087177

## Background

Exposure to tobacco smoke is harmful to children, resulting in higher rates of pneumonia, ear infections, sudden infant death syndrome (SIDS), asthma, and numerous other negative health effects [1]. The tobacco smoke exposure (TSE) of children due to parental tobacco use is a serious and prevalent health issue; research conducted over the course of the past decade shows that over 25 percent of children live in a home with at least one parent who smokes, according to parent report [1-4]. In fact, the 2006 Surgeon General’s report on involuntary tobacco smoke exposure shows that over 60 percent of children have measurable levels of exposure to tobacco [1]. Compared to non-smoking adults, they are three times more likely to live with someone who smokes [1]. Data collected in the early 2000s shows that children exposed to tobacco smoke at a young age are more likely to become smokers themselves and continue the cycle of smoking [5]. In multi-unit housing, children can be exposed to smoke, even if no one smokes in their own unit [6].

For many underserved families, the pediatric office may be their most reliable connection with to health care system, as parental smokers often see their child’s doctor more frequently than their own, with an average of over 10 visits in the first two years of a child’s life [7,8]. In a national survey conducted in 2006, less than 40% of smoking parents reported that their child’s clinician advised them to quit smoking [9]. Low rates of tobacco control services may be due to a lack of knowledge of how to address tobacco use and exposure and office systems that do not support routine screening and assistance for parents who smoke. Parents, however, do approve of their pediatrician’s addressing parental smoking and many would accept enrollment in free tobacco quitlines and medications to help them quit [9,10]. Many pediatricians reported lacking tools for the pediatric setting to address tobacco use and the tobacco smoke exposure of children [11]. In the 2006 Ambulatory Pediatric Association Policy on Tobacco, clinician training programs are urged to increase delivery of services in tobacco prevention, cessation, and the reduction of secondhand smoke exposure [12].

To train pediatric health care providers to address tobacco use and the tobacco smoke exposure of children, we have developed a comprehensive training, support, and dissemination system, CEASE (Clinical Effort Against Secondhand Smoke Exposure). CEASE features materials for the that prompt delivery of tobacco control services; training on how to counsel parents on the importance of no-smoking rules in the home and car, and training on effective use of nicotine replacement therapy [13]. The first training efforts for CEASE included a training manual, a one-on-one training phone call with the practice champion, a practice-wide training session for all pediatric health care providers including pediatric office staff over the phone, and/or an in-person training session with the whole staff. Pilot studies have shown that being trained to use CEASE in the pediatric office leads to a ten-fold increase in the delivery of tobacco dependence treatments [14].

However, traditional CEASE training strategies can be resource intensive for both the training staff and the clinicians being trained, especially the coordination of in-person trainings to locations far from the trainers. The aim of this study was to develop and test innovative distance training and materials for clinicians to address the tobacco smoke exposure of children, the establishment of no-smoking rules, and parental smoking cessation. We hypothesized that rates of asking about family tobacco use and advising about home and car smoking rules by clinicians and pediatric office staff (hereafter referred to as pediatric health care providers) would increase significantly after the intervention was delivered in two pediatric community health center practices.

## Methods

### Sample

Two pediatrics departments (known hereafter as offices, as they act as offices within the health centers) from health centers with a primarily Medicaid population in the greater Boston area were recruited to take part in the study. To be eligible for the study, each study site was required to have high speed internet access, to have a minimum of 65 childcare visits per day, and the designated project leader/champion was required to complete the online component of the training. Low income health clinics were selected for the study because their community populations tend to have high smoking rates and reduced access to the adult health care system. The first two pediatric offices from low income health centers that were contacted met the criteria and were interested in participating in the study; a pediatrician from each office agreed to serve as the project leader and act as the main contact person for the study and project training coordinator. While not required for the study, most clinicians in both practices were English/Spanish bilingual.

**Figure 1:**
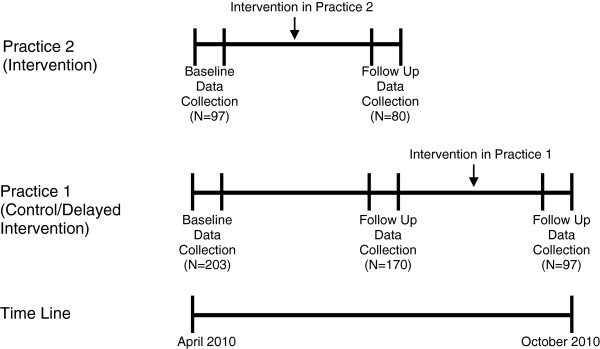
Quasi-experimental study design and intervention timing.

Baseline data was collected for a week at both the practices. The 2 practices were randomized by a coin toss and the intervention was launched in Practice 2 (Intervention practice) and Practice 1 (Control/Delayed intervention practice hereafter referred to as control practice) continued to provide usual care to their patients. Data was again collected 6 weeks post-intervention at both the practices for one week. Practice 1 was then given the intervention and post-intervention data was collected for a week after they had implemented the intervention (see Figure 1). The data was collected from April 2010 to October 2010. The study was approved by the Institutional Review Board of Massachusetts General Hospital.

### The intervention

The distance training and intervention consisted of training pediatric health care providers in pediatric tobacco control and materials to support practice change. The training included a new online training module, telephone training calls, email support, and a training manual, complete with a training DVD. The online portion of the intervention was developed for the study in conjunction with the American Academy of Pediatrics (AAP) online training division. The course, *Help Every Family Quit Smoking*, worth one AAP CME credit, is based on previously tested pediatric practice-based methods to help families quit smoking [15]. Through video clips, educational slides, and quizzes, pediatric clinicians learn about the benefits of parental smoking cessation as well as the harms of tobacco use, tobacco smoke exposure and children, and thirdhand smoke [16]. Clinicians are also coached in how to help families set smoke-free home and car rules and help all family members who smoke quit smoking. The course takes approximately one hour to complete. For the study, the designated project leader/champions completed the online component of the training, but the online training was offered for free to any other physicians or nurses who wished to complete the PediaLink course.

Other aspects of the training included a whole office telephone training call, a training manual, and email/telephone support, which had been previously developed as part of the CEASE intervention to meet the needs of child health care clinicians [17], but were tailored to the needs of the pediatric office being trained and to the distance-based context of the intervention (pediatric offices were asked about the online training and supported with any problems they had with it and were supported by phone and email if there were any questions about the intervention) in general. All clinicians and staff were requested to attend the telephone training call, for which lunch was provided; the call included watching components of the training DVD on the call, asking about how aspects of the intervention could be tailored for the practice, and addressing any potential barriers that practice staff foresaw.

The intervention also included practice support materials, which had been developed for prior CEASE projects; the materials included a one page sheet to ask and document family smoking status and smoke-free home and car rules, educational materials about smoking for families, a fax referral form to the tobacco cessation quitline, posters promoting smoking cessation, and preprinted prescription pads for nicotine replacement therapy. The materials were provided for free and were also available on the CEASE website.

The practice was considered trained in the intervention after the project champion completed the online training module, after the training telephone calls, and after the project champion had completed a 10 chart review to check that smoking status had been documented.

### Data collection

The parent exit survey gathered demographic information: parent’s age, gender, race and ethnicity, and level of education; the age of the youngest child present at the visit; and how the visit was paid. Parents were asked a series of questions to determine if smoking behaviors or policies were discussed by any pediatric health care provider during their visit at the pediatric office: “At any time in your visit today did anyone ask if you”: (1) smoke cigarettes; (2) have a smoke-free home; or (3) have a smoke-free car. Eligible parents read and signed informed consent forms, approved by Massachusetts General Hospital, before completing the survey. In addition, enrolled parents were asked if during their visit their child’s health care provider advised them to: (1) stop smoking; (2) have a smoke-free home; or (3) have a smoke-free car. The majority of the measures were culled from previously validated health and tobacco control survey instruments, including the Behavioral Risk Factor Surveillance System [18] and the Social Climate Survey of Tobacco Control [19]. Experimental questions included items about exact services that the pediatric office provided, such as handing out materials and enrolling in the tobacco control quitline.

A bilingual research assistant unknown to the participants conducted spoken exit interviews over a one week period pre and post intervention with eligible parents (over 18, Spanish or English speaking, with a child seen at the practice that day). While some in the practice knew that the research assistant was at the practice, this fact was true at both baseline and follow-up time points. In addition, many efforts were taken to reduce bias, such as having the research assistant conduct interviews in the most private location possible, away from the reception desk or other areas where clinicians could hear and be reminded of the presence of the research assistant. Though both practices attested that the practice saw a minimum of 65 childcare visits per day, it was quickly learned that the practices did not consistently see this number of patients due to factors such as school holidays, summer vacations, and staff changes. The research assistant attempted a complete capture of exiting parents.

### Data analysis

The primary outcome for this study was a comparison of rates of pediatric health care provider’s asking and advising parents about smoking and smoke-free home and cars at 6 weeks post-intervention in intervention and control practices, as assessed by exit interviews of parents. We present descriptive statistics on the demographics and smoking behavior of the participants. We used chi-squared tests to compare post-intervention changes in the pediatric health care providers’ rates of asking and advising with smoking and smoke-free home and cars in both the control and intervention practice. Further, we also compared the rates of clinician’s asking and advising parents about smoking and smoke-free home and cars pre and post intervention. Since the sample size for individual practices was small, we combined the pre intervention and post intervention data respectively of both the practices for this analysis. All analyses were conducted using Stata statistical software (StataCorp, 2008. Stata Statistical Software: Release 10. College Station, TX: Stata Corporation).

## Results

In Practice 1, a total of 470 parents were interviewed over 3 one-week time periods (203 and 170 parents in each of the two baseline/pre-intervention weeks and 97 parents in the one post-intervention week; in Practice 2, 177 parents were interviewed over 2 one-week time periods (97 parents in the baseline/pre-intervention week and 80 parents in post-intervention week). Table 1 shows the demographic characteristics of both Practice 1 and Practice 2. As expected for such health clinics, the majority of families were insured through Medicaid (80% in control, 70% in intervention). Most selected Hispanic/Latino as their ethnicity (85% in control, 74% in intervention); in the control practice, the majority of survey respondents answered the survey in Spanish (75%), while in the intervention practice, English was the language of choice (77%).

**Table 1:** Demographic characteristics of parents in Pedialink-CEASE

**Variable**	**Practice 1(Control)**	**Practice 2 (Intervention)**	**P-value**
	**N (%)**	**N (%)**	
	**Total N = 470**	**Total N = 177**	
**Age**			0.144
18-24	69 (14.9)	36 (20.5)	
25-44	357 (76.9)	120 (68.2)	
45-64	37 (8)	19 (10.8)	
≥65	1 (0.2)	1 (0.6)	
**Gender**			0.692
Male	78 (16.6)	26 (14.7)	
Female	391 (83.2)	151 (85.3)	
**Relation to child**			0.057
Mother	380 (80.9)	139 (78.5)	
Father	76 (16.2)	25 (14.1)	
Other	13 (2.8)	13 (7.3)	
**Insurance status**			0.056
Medicaid	380 (80.9)	125 (70.6)	
Private insurance/HMO	78 (16.6)	48 (27.1)	
Self pay	8 (1.7)	4 (2.3)	
Other	2 (0.4)	0 (0.00)	
**Education**			0.000
Less than high school	198 (42.1)	26 (14.7)	
High school graduate/GED	163 (34.7)	60 (33.9)	
Some college	49 (10.4)	34 (19.2)	
Trade school	12 (2.6)	6 (3.4)	
College Graduate	41 (8.7)	32 (18.1)	
Graduate or professional school	6 (1.3)	18 (10.2)	
**Race/Ethnicity**			0.000
Hispanic/Latino	401 (85.3)	132 (74.6)	
Non-Hispanic White	44 (9.3)	16 (9.0)	
Non-Hispanic Black	10 (2.1)	21 (11.9)	
Non-Hispanic Asian	6(1.3)	3 (1.7)	
Non-Hispanic Other	3 (0.6)	4 (2.2)	
**Owns a car**			0.376
Yes	266 (56.6)	107 (60.5)	
No	204 (43.4)	70 (39.6)	
**Language of Survey**			0.000
Spanish	351 (74.7)	41 (23.2)	
English	119 (25.3)	136 (76.9)	

At 6 weeks post-intervention in the intervention practice, as compared to the control practice (Table 2), we saw significantly increased rates of parental reports of being screened about: 1) self smoking (22% vs. 41%, p = 0.002), 2) whether a household member smokes (26% vs. 48%, p = 0.001) and 3) smoke-free home (25% vs. 44%, p = 0.008).

**Table 2:** Practice 1 (Control) vs. practice 2 (6 weeks post-intervention)

**Variable**	**Practice 1**	**Practice 2**	**p-value**
	**(Control)N = 170**	**(Intervention) 6 weeks post-Intervention N = 80 n (%)**	
	**n (%)**		
Asked if smoke cigarettes	38 (22)	33 (41)	0.002*
Asked if household member smokes	44 (26)	38 (48)	0.001*
Asked if smoke-free home	42 (25)	35 (44)	0.008*
Asked if smoke-free car	23 (14)	11 (14)	0.962
Advised to have smoke-free home	34 (20)	15 (19)	0.816
Advised to have smoke-free car	24 (14)	11 (14)	0.938

*Statistically significant.

Overall, we had baseline/pre-intervention data for 470 parents (combined 2 weeks baseline/pre-intervention data from practice 1 and 1 week pre-intervention data from practice 2) and post-intervention data for 177 parents (combined 1 week post-intervention data from practice 1 and practice 2 each). The overall combined pre-post intervention data showed statistically significant increased overall rates of parental reports of being asked by a pediatric health care provider about smoking (24% vs. 39%, p < 0.001), smoke-free home (24% vs 38%, p = 0.000) and car rules (11% vs. 22%, p < 0.001), and asking whether a household member smokes (30% vs. 43%, p = 0.002), as can be seen in Table 3. Overall, we also saw a significant increase in pediatric health care providers advising parents to have a smoke-free car post-intervention.

**Table 3:** Overall rates of change in ask and advise pre and post intervention

**Variable**	**Overall rate of change in ask and advise**		
	**Pre-intervention (Practice 1 & 2 combined)**	**Post-intervention (Practice 1 & 2 combined)**	**p-value**
	**N = 470**	**N = 177**	
	**n (%)**	**n (%)**	
Asked if smoke cigarettes	113 (24%)	70 (39%)	P < 0.001*
Asked if household member smokes	140 (30%)	76 (43%)	0.002*
Asked if smoke-free home	115 (24%)	68 (38%)	P < 0.001*
Asked if smoke-free car	53 (11%)	39 (22%)	P < 0.001*
Advised to have smoke-free home	80 (17%)	36 (20%)	0.327
Advised to have smoke-free car	53 (11%)	31 (18%)	0.035*

*Statistically significant.

## Discussion

In this study, we demonstrated that a completely distance-based tobacco control education intervention can improve screening rates of asking families about tobacco use and smoke-free home and car rules by the clinicians and staff in the pediatric departments of these health clinics.

Through the training and intervention materials, the pediatric offices of the health clinics at least partially implemented effective techniques to screen about child’s exposure to tobacco smoke using a standardized question: “does your child live with anyone who uses tobacco?”. Overall, the pediatric offices also improved in rates of advising parents to have a smoke-free car. The implementation of the distance-based intervention represents a noteworthy change from the widespread, non-systematic methods for addressing parental tobacco use in the child health care setting.

This modest initial implementation was accomplished in urban pediatric clinics with a primary Medicaid population, comprised of some of the most vulnerable members of society; this is a historically difficult setting and population to effect service improvement without the addition of staff or significant resources. Increasing the rates of advising parents to have strict smoke-free home and car rules, especially where parents may not be ready to quit smoking, can protect children from the harms of tobacco smoke exposure, and represents an important first step in changing office practices to address parental tobacco use in a routine manner. In homes where parents do not smoke, children are protected from smoking visitors by strict smoke-free rules.

Smoking in cars has recently been emphasized for intervention in child health care settings. Cars are enclosed spaces where children have no escape from breathing tobacco smoke. Children can be exposed to tobacco smoke, even after the cigarette is out [20]. Children who ride in smoke-free cars are protected from harmful off-gassing of tobacco toxins, also known as thirdhand smoke. Parents who believe that thirdhand smoke is harmful to children’s health are more likely to have smoking bans [16], highlighting an important opportunity to for pediatric practices to educate families about the harms of second and thirdhand smoke exposure and assist them in setting completely smoke-free rules at home and in the car.

Interventions that are feasible in the pediatric offices of health centers such as the ones in this study increase access to populations most at risk for tobacco use and exposure. Clinicians at primarily Medicaid health centers are often overworked and have a high turnover rate, due in part to the structure of residency rotations within the health centers. In the study sites, we found that there were very few instances of all clinicians being on site at the same time, as many clinicians worked only a few hours a week. These logistical complications highlight the importance of deploying distance-based training, where each clinician can take the online module at any time.

## Conclusion

Through the distance-based intervention, the trained pediatric clinicians have a great opportunity to address tobacco use and exposure using state of the art approaches. This intervention was feasibly deployed from a distance and documented increased rates of asking about smoking, asking about smoke-free homes, and asking about smoke-free cars, as well as increased rates of advising families to establish smoke-free car rules to protect their children from the harms of tobacco smoke. A completely distance-based tobacco control intervention for pediatrics has the potential to change practice behavior, at least in the short term.

### Limitations

While the research assistant at the practice attempted complete capture of all parents leaving the practice, some parents may have been missed; it is unknown how many parents exiting the practice were missed. As well, the patient flow rate in both practices was much lower than anticipated, due to circumstances beyond the control of the study team or practice.

We do not have data on the sustainability of the intervention nor do we have follow-up data on parent smoking behavior in homes and cars. Through the American Academy of Pediatrics PediaLink division, the CEASE team has developed a more intensive quality improvement module: the eQIPP module, which is a long-term quality improvement training system. The eQIPP module has been developed to lead practices through systematic changes in their practice around addressing tobacco use and exposure. This module, available at http://www.eqipp.org, is currently undergoing rigorous testing and may mitigate implementation issues experienced in this study [21].

## Competing interest

The authors report no competing interest. The authors alone are responsible for writing this article, and for its contents.

## Authors’ contributions

JW and BH conceived the study idea and led the design. All authors provided insight and contributed considerably to the overall study completion. ENB and SR conducted the statistical analysis. BH, ENB, and NH prepared the first draft of the manuscript; the other authors commented on the drafts and provided feedback in the editing process. All authors have read and approved the final manuscript for submission.

## Authors’ note

There are no prior publications or submissions with any overlapping information, including studies and patients. The manuscript has not been and will not be submitted to any other journal while it is under consideration by BMC Pediatrics.

## Pre-publication history

The pre-publication history for this paper can be accessed here:

http://www.biomedcentral.com/1471-2431/13/56/prepub

## References

[B1] U.S. Surgeon General The health consequences of involuntary exposure to tobacco smoke. a report of the Surgeon General 2006 Washington, DC: Department of Health and Human Services 20669524

[B2] Schuster MA Franke T Pham CB Smoking patterns of household members and visitors in homes with children in the United States Arch Pediatr Adolesc Med 2002 156 11 1094 1100 12413336 10.1001/archpedi.156.11.1094

[B3] Soliman S Pollack HA Warner KE Decrease in the prevalence of environmental tobacco smoke exposure in the home during the 1990s in families with children Am J Public Health 2004 94 314 320 10.2105/AJPH.94.2.314 14759948 PMC1448249

[B4] King K Martynenko M Bergman MH Liu YH Winickoff JP Weitzman M Family composition and children's exposure to adult smokers in their homes Pediatrics 2009 123 4 e559 e564 10.1542/peds.2008-2317 19336347 PMC4049446

[B5] Bricker JB Leroux BG Peterson AV Jr Nine-year prospective relationship between parental smoking cessation and children’s daily smoking Addiction 2003 98 5 585 593 10.1046/j.1360-0443.2003.00343.x 12751972

[B6] Wilson KM Klein JD Blumkin AK Gottlieb M Winickoff JP Tobacco-smoke exposure in children who live in multiunit housing Pediatrics 2011 127 85 92 10.1542/peds.2010-2046 21149434

[B7] Klein JD Portilla M Goldstein A Leininger L Training pediatric residents to prevent tobacco use Pediatrics 1995 96 2 Pt 1 326 330 7630693

[B8] Newacheck PW Stoddard JJ Health insurance and access to primary care for children N Engl J Med 1998 338 8 513 519 10.1056/NEJM199802193380806 9468469

[B9] Winickoff JP Tanski SE McMillen RC Hipple BJ Friebely J Healey EA A national survey of the acceptability of quitlines to help parents quit smoking Pediatrics 2006 117 4 e695 e700 10.1542/peds.2005-1946 16585283

[B10] Winickoff JP Tanski SE McMillen RC Klein JD Rigotti NA Weitzman M Child health care clinicians’ use of medications to help parents quit smoking: a national parent survey Pediatrics 2005 115 4 1013 1017 10.1542/peds.2004-1372 15805379

[B11] Winickoff JP Berkowitz AM State of the art interventions for office-based parental tobacco control Pediatrics 2005 115 3 750 760 10.1542/peds.2004-1055 15741382

[B12] Best D Ambulatory pediatric association policy on tobacco Ambul Pediatr 2006 6 6 332 336 10.1016/j.ambp.2006.09.002 17116606

[B13] Winickoff JP “The clinical effort against secondhand smoke exposure (CEASE) intervention: a decade of lessons learned” JCOM 2012 19 9 PMC3874254 24379645

[B14] Winickoff JP Friebely J Healey E Addressing parental smoking by changing pediatric office systems 2007 Austin TX: Presented at the Society for Research on Nicotine and Tobacco

[B15] Winickoff JP Park ER Hipple BJ Clinical effort against secondhand smoke exposure: development of framework and intervention Pediatrics 2008 122 2 e363 e375 10.1542/peds.2008-0478 18676523 PMC2774730

[B16] Winickoff JP Friebely J Tanski SE Sherrod C Matt GE Hovell MF Beliefs about the health effects of “thirdhand” smoke and home smoking bans Pediatrics 2009 123 e74 e79 10.1542/peds.2008-2184 19117850 PMC3784302

[B17] Hall N Addressing family smoking in child health care settings J Clin Outcomes Manag 2009 16 8 367 373 20448841 PMC2864638

[B18] The behavioral risk factor surveillance system (BRFSS) Available at http://www.cdc.gov/brfss/

[B19] The social climate survey of tobacco control (SCS-TC) Available at http://www.socialclimate.org/

[B20] Matt GE Quintana PJ Hovell MF Households contaminated by environmental tobacco smoke: sources of infant exposures Tob Control 2004 13 1 29 37 10.1136/tc.2003.003889 14985592 PMC1747815

[B21] Winickoff J EQIPP: eliminate tobacco Use and exposure http://www.eqipp.org

